# Perioperative and Oncological Outcome in Patients Undergoing Curative-Intent Liver Resection for Cholangiocarcinoma in the Context of Osteopenia

**DOI:** 10.3390/cancers17132213

**Published:** 2025-07-01

**Authors:** Franziska A. Meister, Katharina Joechle, Philipp Tessmer, Esref Belger, Anjali A. Roeth, Oliver Beetz, Felix Oldhafer, Jan Bednarsch, Ulf P. Neumann, Carolin V. Schneider, Robert Siepmann, Iakovos Amygdalos, Florian W. R. Vondran, Zoltan Czigany

**Affiliations:** 1 Department of General, Visceral, Pediatric and Transplantation Surgery, University Hospital RWTH Aachen, 52074 Aachen, Germany; kjoechle@ukaachen.de (K.J.); ptessmer@ukaachen.de (P.T.); ebelger@ukaachen.de (E.B.); aroeth@ukaachen.de (A.A.R.); obeetz@ukaachen.de (O.B.); foldhafer@ukaachen.de (F.O.); iamygdalos@ukaachen.de (I.A.); fvondran@ukaachen.de (F.W.R.V.); zoltan.czigany@med.uni-heidelberg.de (Z.C.); 2 Department of General, Visceral and Transplantation Surgery, University Hospital Essen, 45147 Essen, Germany; jan.bednarsch@uk-essen.de (J.B.); ulf.neumann@uk-essen.de (U.P.N.); 3 Department for Gastroenterology, Metabolic Diseases and Intensive Care, University Hospital RWTH Aachen, 52074 Aachen, Germany; cschneider@ukaachen.de; 4 Department of Diagnostic and Interventional Radiology, University Hospital RWTH Aachen, 52074 Aachen, Germany; rsiepmann@ukaachen.de; 5 Department of General, Visceral and Transplantation Surgery, University Hospital Heidelberg, 69120 Heidelberg, Germany

**Keywords:** CCA, body composition, osteopenia, bone mineral density, liver resection

## Abstract

**Background**: Cholangiocarcinoma (CCA) of the liver is a highly aggressive cancer that arises from malignant cells in the bile ducts. Radical surgery remains the only curative option, but major liver resection carries high perioperative risks. This study investigates the predictive value of preoperative bone mineral density (BMD), measured via CT, for perioperative complications, mortality, and long-term outcomes. **Methods**: The analysis included 202 patients who underwent curative-intent surgery for intrahepatic cholangiocarcinoma (iCCA; n = 97) or perihilar cholangiocarcinoma (pCCA; n = 105) between 2010 and 2019. Preoperative bone mineral density (BMD) was assessed using computed tomography segmentation at the level of the 12th thoracic vertebra. Osteopenia was defined according to established cutoffs. **Results**: Osteopenia was highly prevalent in both iCCA (53/97, 54%) and pCCA (54/105, 51%) subcohorts. Patients suffering from osteopenia were significantly older than those without (71.1 [62–76.6] years vs. 61.3 [52.9–69.2] years; *p* < 0.001). Alteration in BMD did not demonstrate a significant prognostic effect in terms of perioperative morbidity (Mann–Whitney U; comprehensive complication index—CCI: 34 [9–56] vs. 40 [21–72] *p* = 0.185; iCCA: *p* = 0.803; pCCA: *p* = 0.165). The median overall survival in our cohort was 19 [14–25] months. Patients with osteopenia did not exhibit a significantly different overall survival compared to those with normal bone mineral density (log-rank *p* = 0.234). **Conclusions**: In contrast to our previous observations in other oncological patient cohorts, osteopenia alone had no significant negative impact on clinical outcomes in our large European cohort of patients undergoing curative-intent surgery for CCA. To validate these findings, further prospective studies are warranted.

## Simple Summary

Cholangiocarcinoma is a rare but aggressive form of liver cancer that starts in the bile ducts. Surgery is currently the only chance for a cure, but it carries high risks, especially in older or physically weaker patients. This study explores whether measuring bone mineral density using routine CT scans before surgery could help predict who is at greater risk of complications or poor outcomes as osteopenia is often linked to aging and frailty. We studied over 202 patients who underwent surgery with curative intent and compared the outcomes between those with and without osteopenia. Surprisingly, we found that having low bone density did not lead to worse surgical outcomes or survival rates. These findings suggest that, unlike in other cancer types, osteopenia may not be a reliable prognostic factor for patients undergoing surgery for cholangiocarcinoma. Further research is needed to confirm these results.

## 1. Introduction

In carcinomas with poor prognosis, careful patient selection for surgical treatment is essential to optimize postoperative outcomes. This consideration is particularly relevant in cases such as cholangiocarcinoma (CCA) of the liver, where the aggressive nature of the disease and limited therapeutic options make it critical to identify patients who are most likely to benefit from surgical intervention, the only curative treatment option available to date. As patients requiring major liver resection for CCA are confronted with a substantial risk of perioperative morbidity and mortality, accurate patient selection and risk stratification are crucial in optimizing outcomes.

Impairment of body composition (BC) including reduced muscle mass (sarcopenia) and impaired muscle quality (myosteatosis) has been recognized as a risk factor in various settings [1,2,3,4]. Recent studies, including those from our group, have identified a high prevalence of sarcopenia and myosteatosis in patients with CCA, hepatocellular carcinoma (HCC), or liver cirrhosis [5,6,7,8]. Moreover, these studies have revealed a correlation between myosteatosis and unfavorable perioperative outcomes in patients undergoing partial hepatectomy for CCA, HCC, and orthotopic liver transplantation (OLT) [5,6,8,9].

Recently, Pereira et al. published that bone loss in male patients with chronic diseases may occur and become clinically apparent prior to any noticeable reduction in skeletal muscle mass [10]. While dual-energy X-ray absorptiometry (DXA) remains the gold standard for assessing bone mineral density (BMD), there is a growing trend towards utilizing CT scan-based attenuation values for characterizing BMD. This approach is gaining popularity, particularly in oncological patients, owing to the widespread availability of CT scans as part of preoperative staging [11]. In this context, reduced BMD, defined as osteopenia, has recently been analyzed and could be found to impair oncological outcomes in patients undergoing surgical resection for HCC by our group and others [12,13].

Based on this, Jordens et al. recently identified a correlation between survival and osteopenia in female palliative CCA patients [14], and the group of Watanabe et al. demonstrated a survival disadvantage in osteopenic patients who underwent surgery for pCCA in a Japanese cohort [15]. Nevertheless, there is still a lack of knowledge concerning BMD and its potential prognostic role in patients suffering from CCA. Accordingly, the objective of this study was to investigate the prognostic significance of osteopenia in clinical outcomes among a cohort of patients undergoing partial hepatectomy for intrahepatic cholangiocarcinoma (iCCA) and perihilar cholangiocarcinoma (pCCA) with curative intent at a single center in Western Europe.

## 2. Patients and Methods

### 2.1. Patients and Eligibility

This retrospective study analyzed all consecutive patients who underwent partial hepatectomy for CCA at the University Hospital RWTH Aachen, Germany, between 2010 and 2019. Prior to elective partial hepatectomy, clinical staging was conducted, excluding patients with systemic or unresectable disease. Additionally, those who received only abdominal MRI for staging were not included in the bone mineral density (BMD) analysis. The study adhered to the International Conference on Harmonisation Good Clinical Practice (ICH-GCP) guidelines and the Declaration of Helsinki. The study protocol was approved by the RWTH-Aachen Institutional Review Board (EK 115/20 and EK 341/21). Due to the study’s retrospective design and use of pre-existing clinical data, informed consent was waived.

### 2.2. Image Analysis and Segmentation

**Figure 1:**
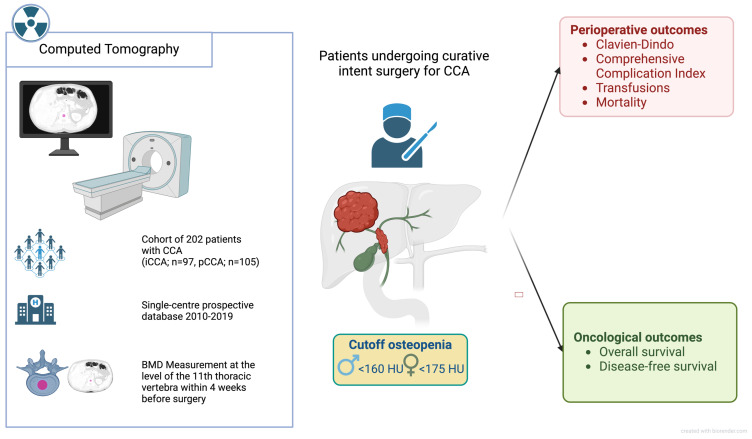
Summary of the study design and analysis approach.

Bone mineral density (BMD) was evaluated using imaging data, following a previously described method that involved analyzing a single cross-sectional image at the level of the 11th thoracic vertebra (Figure 1) [12,16]. Computed tomography for oncological staging was performed at UH-RWTH Aachen within four weeks before surgery. All CTs were performed using a dual-source-CT-scanner (Siemens Somatom Force, Siemens AG, München, Germany) with the following technical parameters: tube voltage, 120 kVp; 0.5 s/rotation; and 5 mm reconstruction thickness. Segmentation was performed in a semi-automated manner by an investigator blinded to the patients’ remaining clinical data using the 3D Slicer software platform version 4.1 (https://www.slicer.org/). This involved calculating the average pixel density within a standardized circular region of interest (ROI), specifically targeting the mid-vertebral core sample on trabecular bone alone, using the non-contrast phase of the CT scans. To mitigate partial volume effects, three standardized, circular regions of interest (ROIs) were manually placed within the trabecular bone of the mid-vertebral body for each patient. The mean of these three measurements was calculated to obtain a robust and representative estimate of vertebral bone mineral density (BMD). Patients with radiodense foreign materials (e.g., spinal implants or cement) that could induce beam-hardening artifacts were excluded to minimize measurement bias. The resulting BMD values were measured in Hounsfield units (HU). A gender-specific cutoff value was used to define osteopenia. While for male patients, a pre-established cutoff value of <160 HU was determined, a cutoff of <175 HU was defined for females, as described previously [12].

### 2.3. Clinical Data Collection and Patient Follow-Up

Clinical data were retrospectively analyzed from a prospectively maintained institutional database. The decision to pursue a curative-intent partial hepatectomy was made by a hepatobiliary surgeon and later approved by the interdisciplinary tumor board at the institution. Partial hepatectomy procedures were conducted either laparoscopically or conventionally as described in earlier studies of our group [12]. Follow-up data utilized in this study were sourced from the outpatient clinic of the UH-RWTH Aachen and the local community-based oncologist network. The classifications and scores utilized in this analysis have been elucidated in prior published studies, both by our group and others (including ASA, labMELD, Clavien–Dindo classification (CD) and the comprehensive complication index (CCI) [17,18], calculation of blood transfusion, length of hospital stay, and long-term follow-up).

### 2.4. Statistical Analysis

The primary endpoint of this study was to evaluate the overall survival (OS) of patients who underwent liver resection for CCA with curative intent. Secondary endpoints included the assessment of perioperative in-hospital major morbidity (defined as CD ≥ 3b) [17], overall perioperative outcome, length of hospital stay, 90-day mortality, and disease-free survival (DFS). DFS was defined as the time from curative-intent surgery to cancer recurrence, while death from other causes than the primary cancer was censored. OS was defined as the time from curative-intent surgery to death from any cause. Categorical data were presented as absolute and relative frequencies, while continuous data were expressed as the mean ± standard deviation. Statistical analysis involved the use of the Chi-square test and Fisher’s exact test for categorical data, and the Student’s *t*-test, Mann–Whitney U test, and Kruskal–Wallis H test for continuous data. Spearman’s correlation coefficient was utilized to examine the association between BMD and various BC parameters. Kaplan–Meier survival curves were generated and analyzed using the log-rank test. Figures were created with GraphPad Prism 9 (Dotmatics, Boston, MA, USA), and statistical analyses were conducted using SPSS Statistics 24 (IBM Corp., Armonk, NY, USA), with a significance threshold of *p* < 0.05.

## 3. Results

### 3.1. Study Population Characteristics

Between 2010 and 2019, 225 consecutive patients underwent curative-intent partial hepatectomy for CCA at our university hospital. Twenty-three patients were excluded from the study due to inadequate preoperative imaging, resulting in a final study cohort of 202 patients. Among these, there were 116 male patients (57%) and 86 female patients (43%), with a median age of 66.6 [57.7–73.4] years. A total of 97 (48%) patients were suffering from iCCA, while 105 (52%) patients were diagnosed with pCCA.

### 3.2. Body Composition Assessment and Patient Characteristics

In our cohort, the median BMD was 155 [129.9–187.4] with a median BMI of 25.1 [22.5–28.9]. Based on our pre-defined cutoffs, a total of 107 patients were diagnosed with osteopenia and 95 patients with normal BMD with a median value of 132 [107–143] HU, compared to 189 [176–215] HU (Table 1, respectively).

The osteopenia group had a significantly higher median age of 71.1 [62.0–76.6] years compared to 61.3 [52.9–69.2] years in the non-osteopenia group (*p* = 0.001, Table 1, respectively). The median body mass index (BMI) was similar between the groups: 25.0 [22.1–29.1] kg/m^2^ in the osteopenic group and 25.1 [22.9–28.7] kg/m^2^ in the non-osteopenic group (*p* = 0.921, Table 1, respectively). Interestingly, skeletal muscle mass (SMI) was similar in both groups, while skeletal muscle radiation attenuation (SM-RA) was significantly lower in osteopenic patients (46 [41–53] vs. 48 [42–56]; *p* = 0.325; 31 [25–36] vs. 35 [29–40]; *p* = 0.001, Table 1, respectively)

**Figure 2:**
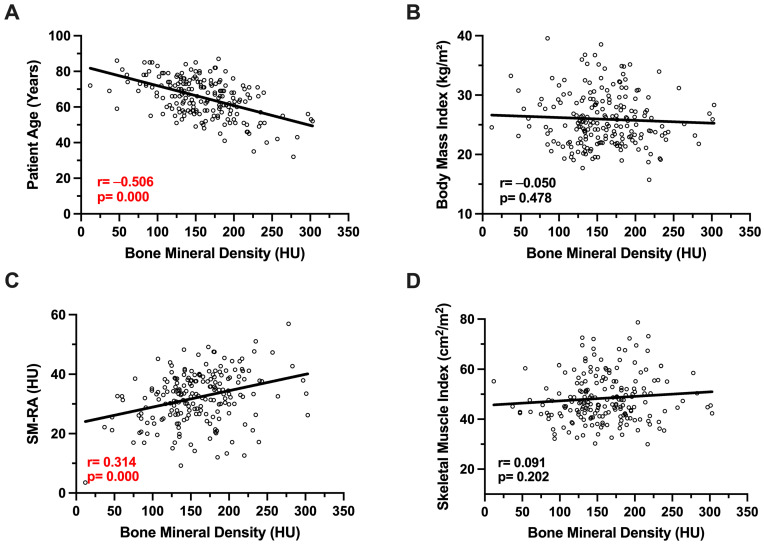
Correlation between bone mineral density and patient age (**A**), body mass index (**B**), muscle density (**C**), and muscle mass (**D**).

In line with these findings, patient age was significantly negatively associated with BMD using Spearman‘s correlation coefficient and corresponding correlations plots, while BMI was not (r = -0.506, *p* = 0.000; r = -0.050, *p* = 0.478, Figure 2). A significant correlation between SM-RA and BMD could be detected, while BMD and SMI showed no association (r = 0.314, *p* = 0.000; r = 0.091, *p* = 0.202, Figure 2).

There were no significant differences between the groups in preoperative characteristics, including ASA classification, preoperative cholangitis, and cirrhosis.

Preoperative serum bilirubin levels were comparable between groups, with a median of 0.70 [0.43–1.35] vs. 0.83 [0.45–2.47] mg/dL (*p* = 0.878, Table 1, respectively). However, preoperative serum gamma glutamyltransferase levels (GGT) were slightly elevated in patients suffering from osteopenia, with a median of 245 [99–630] U/L vs. 246 [120–621] U/L in patients with normal BMD (*p* = 0.041, Table 1, respectively). In 27% of the cohort, preoperative portal vein embolization was performed for 33% of osteopenic patients and 21% of patients with a normal BMD distribution (*p* = 0.081, Table 1, respectively).

### 3.3. Surgical Approach

Regarding the surgical approach, there were no significant differences between the groups in liver resection procedures. Extended right hepatectomies (15%) and extended left hepatectomies (17%) were the most frequently performed procedures (Table 1). Tumor staging was similarly distributed across both groups, with no notable differences in UICC stages, T/N categories, or tumor grading (detailed characteristics in Table 1). R0 resection was achieved in 85% of patients (Table 1).

### 3.4. Perioperative Outcome and Osteopenia

Concerning perioperative outcomes, no statistically significant difference was detected between the osteopenic and non-osteopenic subcohorts. Major postoperative complications (CD ≥ 3b) occurred in 35% of the total cohort: 32% in osteopenic patients and 39% in patients with normal BMD, showing no significant difference (*p* = 0.262, Table 2). The comprehensive complication index (CCI) was marginally higher in the non-osteopenic group, with a median of 39.5 [20.9–72.2] compared to 33.5 [8.7–56] in the osteopenia group, but the difference was not statistically significant (*p* = 0.128, Table 2, respectively). The median duration of the operative procedure was 360 [288–438] minutes, with similar durations in both groups (*p* = 0.274, Table 2).

In line with the findings above, median hospital stay did not differ significantly (15 [11–30] vs. 16 [11–26], *p* = 0.266, Table 2, respectively). Intraoperative red blood cell (RBC) transfusion requirements were minimal, with a median of 0 [0–2] units in both groups (*p* = 0.796), while fresh frozen plasma (FFP) transfusion showed no significant difference, with a median of 2 [0–4] units in the osteopenic group and 0 [0–4] units in the non-osteopenia group (*p* = 0.468, Table 2, respectively).

The osteopenic and non-osteopenic groups were further subdivided into patients suffering from iCCA and pCCA cohorts. Similarly, no significant differences were observed in perioperative outcomes between the groups including the duration of the operative procedure, hospital stay, transfusion requirements, and postoperative complications. Detailed results can be found in Table 2.

### 3.5. The Effect of Osteopenia on Long-Term Overall and Disease-Free Survival

**Figure 3:**
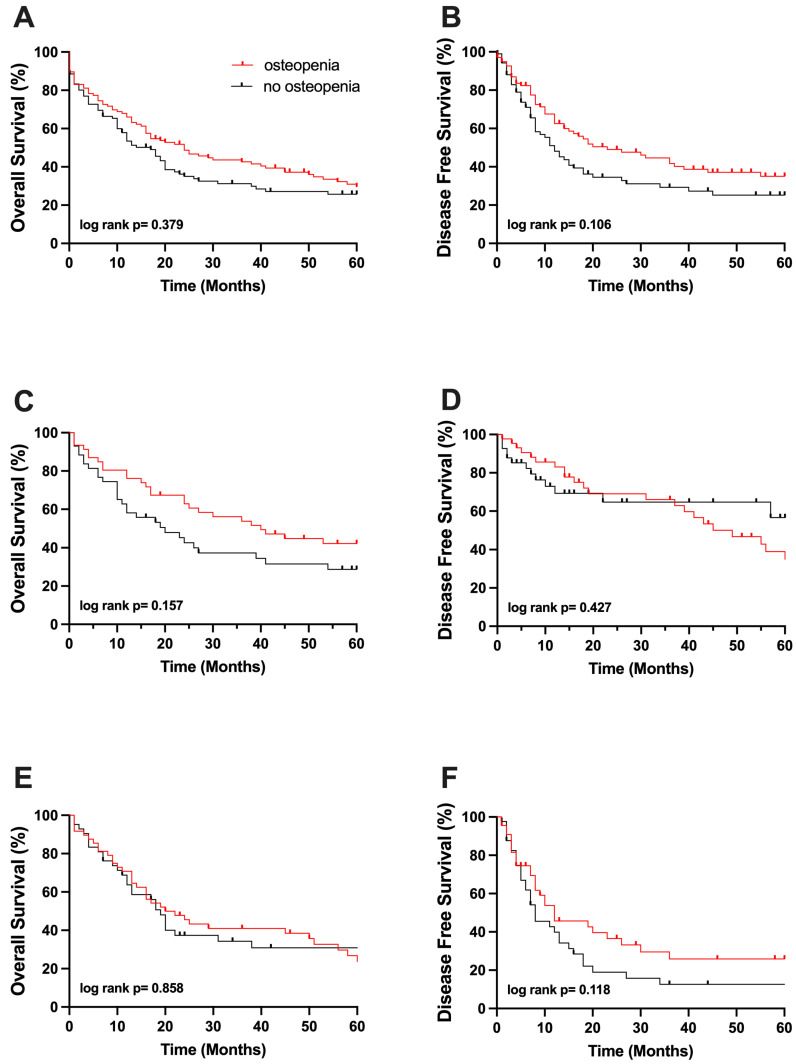
(**A**) Five-year survival of osteopenic and non-osteopenic patients. (**B**) Disease-free survival of osteopenic and non-osteopenic patients. (**C**) Five-year survival of osteopenic and non-osteopenic patients suffering from pCCC. (**D**) Disease-free survival of osteopenic and non-osteopenic patients suffering from pCCC. (**E**) Five-year survival of osteopenic and non-osteopenic patients suffering from iCCC. (**F**) Disease-free survival of osteopenic and non-osteopenic patients suffering from iCCC.

The median overall survival (OS) of all included patients in this study was 19 [14–25] months with a DFS of 16 [11–21] months. Patients with osteopenia had an OS of 24 months, compared to 14 months in non-osteopenic patients. The five-year OS probability of osteopenic patients was similar when compared to those with normal BMD (30% vs. 26%, *p* = 0.379; Figure 3, respectively). In line with the findings above, the probability of DFS at 5 years did not differ significantly between the groups (35% vs. 25%, *p* = 0.106; Figure 3, respectively). Additionally, the group was subdivided into iCCA and pCCA patients. No significant difference in the OS or DFS probabilities at 5 years could be found in osteopenic and non-osteopenic patients suffering from iCCA as well (23% vs. 31%, *p* = 0.858, 26% vs. 13%, *p* = 0.118, Figure 3, respectively). Similar, no significant impact of BMD could be observed concerning the 5-year OS and DFS probabilities in patients with pCCA (42% vs. 29%, *p* = 0.157, 35% vs. 57%, *p* = 0.427, Figure 3, respectively).

Further, due to the sex-related differences in BMD values, we performed a subgroup analysis based on gender. Out of the 116 male patients with CCA, 72 patients had pCCA and 44 iCCA, while the female cohort included 86 patients, 33 with pCCA and 53 with iCCA (Supplementary Table S1).

**Figure 4:**
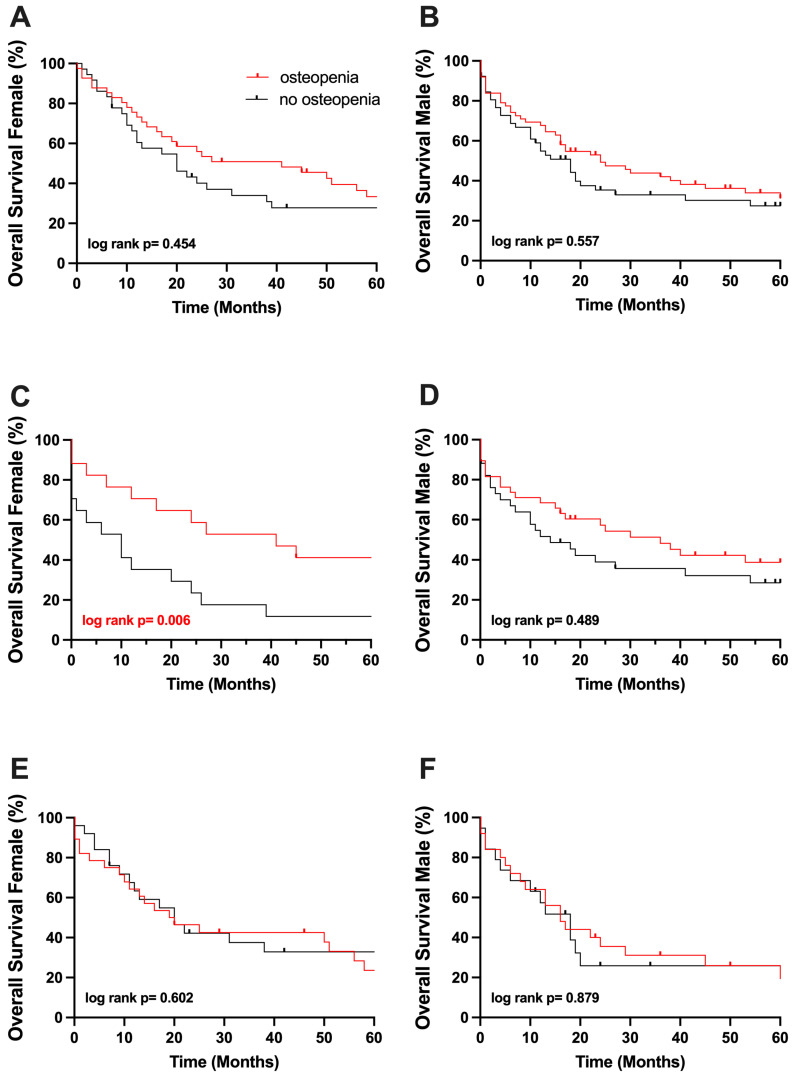
(**A**) Five-year survival of osteopenic and non-osteopenic female patients. (**B**) Five-year survival of osteopenic and non-osteopenic male patients. (**C**) Five-year survival of osteopenic and non-osteopenic female patients suffering from pCCC. (**D**) Five-year survival of osteopenic and non-osteopenic male patients suffering from pCCC. (**E**) Five-year survival of osteopenic and non-osteopenic female patients suffering from iCCC. (**F**) Five-year survival of osteopenic and non-osteopenic male patients suffering from iCCC.

In male patients, 5-year OS probability was comparable in the osteopenic and non-osteopenic groups (31% vs. 28%, *p* = 0.557; Figure 4, respectively). Osteopenic and normal BMD groups did not differ significantly concerning 5-year OS, neither in male patients suffering from pCCA nor in male patients suffering from iCCA (39% vs. 29%, *p* = 0.489, 19% vs. 26%, *p* = 0.879; Figure 3, respectively).

In line with the findings above, osteopenia did not affect OS probability in female patients significantly, with a 5-year OS probability of 33% in osteopenic females and 28% in non-osteopenic females (*p* = 0.454; Figure 4).

Interestingly, in the female subcohort of patients suffering from pCCA, an impaired OS probability at 5 years in non-osteopenic patients could be detected (44% vs. 14%, *p* = 0.006; Figure 4, respectively) notably in a small cohort of 33 individuals, while the 5-year OS probability in females with iCCA was largely comparable (24% vs. 33%, *p* = 0.557; Figure 4, respectively).

## 4. Discussion

The present study aimed to explore the potential association between BMD and both perioperative outcomes and long-term survival in patients undergoing surgery for CCC. Despite the known implications of BMD in clinical outcomes indicated by our group and others in different entities [12,14,19], our present findings suggest that BMD does not have a significant correlation with either the perioperative outcomes or the long-term prognosis in our CCA cohort. Although the subcohort of female patients with pCCA and normal bone density exhibited a shorter overall survival, we hypothesize that the cohort size of female pCCA patients (n = 34) was too small to draw definitive conclusions.

Considering that a correlation between osteopenia and unfavorable outcomes in patients undergoing partial hepatectomy for hepatocellular carcinoma (HCC) could be detected by our group and others [12,13], it is particularly surprising that a similar association could not be found in patients suffering from CCA and thus undergoing surgery with curative intent. In HCC, lower BMD has been linked to impaired clinical and oncological outcomes, possibly due to the shared metabolic and systemic factors, such as chronic liver disease and inflammation, that affect both bone health and tumor progression. The absence of this correlation in CCA might be explained by the underlying liver-related mechanisms: HCC typically develops with a background of liver cirrhosis; in contrast, cholangiocarcinoma does not arise from cirrhotic liver tissue.

Another possible explanation for the lack of association between BMD and outcomes in cholangiocarcinoma could be the highly aggressive and rapidly progressing nature of this tumor. Cholangiocarcinoma often advances quickly, leading to significant clinical deterioration regardless of underlying bone health. This aggressive behavior might overshadow any potential impact that BMD could have on patient outcomes, with the rapid tumor progression being the primary driver of both perioperative and long-term results. In contrast to hepatocellular carcinoma, where the disease course can be more indolent and chronic conditions such as liver cirrhosis can simultaneously affect bone health and cancer progression, ultimately deciding the fate of the patient, cholangiocarcinoma’s swift advancement may leave less room for BMD to play a significant clinical role in influencing patient outcomes. In this context, future research should possibly prioritize the development of preventive measures and alternative treatments for this aggressive cancer, given its rapid progression and poor prognosis.

BMD is widely recognized as the primary parameter for assessing bone mass loss and serves as a key morphological indicator of patient frailty [20]. While DXA remains the gold standard for diagnosing osteopenia and osteoporosis, increasing evidence supports the use of radiation attenuation values from trabecular bone in routine staging CT scans for oncological patients [21,22,23]. While Watanabe et al. defined osteopenic patients using a non-gender-specific pre-described cutoff of <160 HU [15], the group of Jordens et. al. recently examined BMD and its prognostic role in palliative patients suffering from CCA, finding a significant correlation in female patients only [14]. However, they used a median-based gender-specific cutoff for BMD. Similarly, to address well-documented gender differences in BMD values, our study adopted sex-specific cutoffs for osteopenia, aligning with the approach recently employed by Sharshar et al. in a Japanese cohort of patients with pancreatic cancer [21,24]. While we used the well-established and frequently described cutoff of 160 HU for men, the cutoff for women was determined at 175 HU based on findings from a recent HCC cohort [13].

Recent studies have established a link between reduced muscle mass, or sarcopenia, and bone mineral density (BMD). For instance, Szulc et al. found that sarcopenia was connected to thinner bone cortices and an increased risk of falls in older men [25]. However, our study did not confirm a relationship between sarcopenia and skeletal muscle index (SMI). Instead, we identified a highly significant correlation between BMD and muscle density or radiation attenuation, and as expected, a significant negative correlation with patient age.

In our cohort, although patients with osteopenia were significantly older, no increased perioperative risk or adverse long-term survival were observed. These results imply that the presence of osteopenia should not automatically preclude older patients from undergoing surgical interventions. Consequently, it appears that partial hepatectomy for CCA is both safe and effective even for the demographic group of elderly surgical candidates, as was recently reported by Weigle et al. and Gupta et al. [26,27]. This could be an important lesson for countries and HPB programs where advanced age alone is seen as an important limitation for surgery.

Several limitations of this study should be noted. First, there is a need to evaluate whether the osteopenia cutoffs used in our analysis were appropriate for accurately identifying patients at risk for poor outcomes. Factors such as age, sex, race, and other cohort-specific variables can significantly influence BMD values and their distribution. Secondly, the preoperative CT images used for BMD measurement were obtained at different time points during routine clinical practice and analyzed retrospectively without standardized controls.

Despite these limitations, this study represents the first analysis to investigate the potential correlation between osteopenia and clinical outcomes following curative-intent liver surgery for cholangiocarcinoma in a large Western European single-center cohort. Additional prospective clinical trials are required to confirm these findings.

## 5. Conclusions

In this retrospective cohort study of patients undergoing curative-intent resection for intrahepatic and perihilar cholangiocarcinoma, preoperative osteopenia—despite being highly prevalent—did not emerge as a significant predictor of perioperative morbidity, postoperative mortality, or overall survival. This contrasts with prior findings in other cancer populations, where low bone mineral density has been associated with poorer outcomes. While osteopenic patients in our cohort were significantly older, reduced BMD alone did not translate into worse short- or long-term clinical trajectories. These findings suggest that osteopenia, as measured by CT at the thoracic vertebra level, may have limited utility as a standalone prognostic marker in the context of cholangiocarcinoma surgery. Given the complex interplay between age, frailty, and oncologic outcomes, further prospective studies are warranted to explore whether BMD could still contribute to a broader preoperative risk stratification framework.

## Figures and Tables

**Table 1:** Patient and procedural characteristics.

Characteristics	All Patients	Osteopenia		*p*-Value
	n = 202	Yes n = 107	No n = 95	
**Age (years)**	66.6 [57.7–73.4]	71.1 [62–76.6]	61.3 [52.9–69.2]	**0.001**
**BMI (kg/m^2^)**	25.1 [22.5–28.9]	25 [22.1–29.1]	25.1 [22.9–28.7]	0.921
**Bone mineral density (HU)**	155 [129.9–187.4]	132 [107–143]	189 [176–215]	**0.001**
**SMI (cm^2^/m^2^)**	48 [42–55]	46 [41–53]	48 [42–56]	0.325
**SM-RA (HU)**	35 [29–40]	31 [25–36]	35 [29–40]	**0.001**
**Sex ratio (F:M), n (%)**	86 (43):116 (57)	44 (41):63 (59)	42 (44):53 (56)	0.658
**ASA**				0.251
1	8 (4)	2 (3)	6 (6)	
2	81 (40)	41 (38)	40 (42)	
3	100 (50)	59 (55)	41 (43)	
4	9 (5)	5 (5)	4 (4)	
**EBD (stent), n (%)**	100 (50)	55 (51)	45 (47)	0.576
**PBD, n (%)**	24 (12)	13 (12)	11 (12)	0.922
**Cholangitis preoperative**	55 (27)	35 (33)	20 (21)	0.865
**Steatosis**	37 (18)	15 (14)	22 (23)	0.068
**Cirrhosis**	8 (4)	3 (3)	5 (5)	0.590
**Serum CA 19-9 pre-op**	58 [22–232]	64 [19–214]	57 [23–283]	0.653
**Serum Hgb pre-op**	12.8 [11.8–13.8]	12.6 [11.7–13.9]	13 [11.9–13.7]	0.614
**Preoperative AST (U/L)**	41 [30–64]	37 [30–55]	47 [32–82]	0.913
**Serum bilirubin**	0.74 [0.44–1.76]	0.7 [0.43–1.35]	0.83 [0.45–2.47]	0.878
**Preoperative GGT (U/L)**	246 [110–626]	245 [99–630]	246 [120–621]	**0.041**
**Preoperative albumin (g/L)**	41 [36–44]	40 [35–44]	41 [36–44]	0.368
**Portal vein embolization (PVE), n (%)**	55 (27)	35 (33)	20 (21)	0.081
**Laparoscopic approach n (%)**	8 (4)	2 (2)	6 (6)	0.268
**Lymphadenectomy, n (%)**	193 (96)	104 (96)	89 (93)	0.871
**Vascular reconstruction n (%)**	152 (75)	83 (77)	70 (74)	0.830
**Operative Procedure**				0.078
Atypical resection	9 (5)	3 (3)	6 (6)	
Right hepatectomy	26 (13)	10 (9)	16 (17)	
Left hepatectomy	25 (12)	18 (17)	7 (7)	
Extended right hepatectomy	30 (15)	17 (16)	13 (14)	
Extended left hepatectomy	35 (17)	13 (12)	22 (23)	
Right trisectorectomy	27 (13)	19 (18)	8 (8)	
Left trisectorectomy	14 (7)	9 (8)	22 (23)	
Bisegmentectomy	6 (3)	4 (4)	2 (2)	
Hepatoduodenectomy	9 (5)	3 (3)	6 (6)	
ALPPS	14 (7)	7 (7)	7 (7)	
**Tumor Stage UICC (8th edition)**				0.977
0	2 (1)	1 (1)	1 (1)	
I	24 (12)	15 (14)	9 (10)	
II	67 (33)	39 (36)	28 (30)	
III	79 (39)	41 (38)	38 (40)	
IV	20 (10)	9 (9)	11 (13)	
**T category, n (%)**				0.757
Tis	2 (1)	1 (1)	1 (1)	
T1	35	19	16	
T2	119	66	53	
T3	32 (16)	13 (12)	19 (20)	
T4	13 (6)	7 (7)	6 (6)	
**N category, n (%)**				0.056
N0	109 (54)	63 (59)	49 (52)	
N1	67 (33)	37 (35)	30 (32)	
N2	12 (6)	5 (5)	7 (7)	
Nx	9 (5)	1 (1)	8 (8)	
**R category, n (%)**				0.521
R0	150 (74)	76 (71)	74 (78)	
R1	28 (14)	16 (15)	12 (13)	
Rx	19 (9)	12 (11)	7 (7)	
**(Micro** **-** **)vascular invasion, n (%)**	62	31 (29)	31 (33)	0.852
**Lymphovascular invasion, n (%)**	46	22 (21)	24 (25)	0.332
**Perineural invasion, n (%)**	90	40 (37)	50 (53)	0.329
**Tumor grading, n (%)**				0.251
G1	2 (1)	1 (1)	2 (2)	
G2	128 (63)	70 (65)	58 (67)	
G2–3	5 (3)	4 (4)	1 (1)	
G3	50 (25)	25 (23)	25 (26)	
G4	4 (2)	3 (3)	1 (1)	

Values are given as the mean ± standard deviation or numbers and percentages. Abbreviations used: BMI: body mass index; ASA: American Society of Anesthesiologists; AST: aspartate aminotransferase; GGT: gamma glutamyltransferase; BMD (HU): bone mineral density (Hounsfield units); SMI: skeletal muscle index; SM-RA (HU): skeletal muscle radiation attenuation (Hounsfield units); PVE: portal venous embolization; UICC: Union for International Cancer Control.

**Table 2:** Perioperative outcome.

Characteristics	All Patients	Osteopenia		*p*-Value
pCCA + iCCA	n = 202	yes n = 107	no n = 95	
**CD ≥ 3b complications ^1^ including 90-day mortality n (%)**	71 (35)	34 (32)	37 (39)	0.262
**Duration operative procedure (minutes)**	360 [288–438]	355 [277–435]	363 [300–450]	0.274
**Hospital stay (days)**	15 [11–28]	16 [11–26]	15 [11–30]	0.266
**Intraoperative RBC transfusion (units)**	0 [0–2]	0 [0–2]	0 [0–2]	0.796
**Intraoperative FFP transfusion (units)**	2 [0–4]	2 [0–4]	0 [0–4]	0.468
**CCI ^2^**	34.6 [20.9–59]	33.5 [8.7–56]	39.5 [20.9–72.2]	0.128
**pCCA**	**n = 105**	**yes n = 54**	**no n = 51**	
**CD ≥ 3b complications ^1^ including 90-day mortality n (%)**	47 (49)	20 (38)	27 (53)	0.101
**Duration operative procedure (minutes)**	404 [359–474]	390 [358–477]	420 [357–475]	0.479
**Hospital stay (days)**	19 [12–36]	16 [12–36]	20 [13–36]	0.207
**Intraoperative RBC transfusion (units)**	0 [0–2]	0 [0–2]	0 [0–2]	0.535
**Intraoperative FFP transfusion (units)**	3 [0–4]	3 [0–4]	4 [0–5]	0.820
**CCI ^2^**	41 [21–74]	38 [21–60]	30 [0–47]	0.133
**iCCA**	**n = 97**	**yes n = 53**	**no n = 44**	
**CD ≥ 3b complications ^1^ including 90-day mortality n (%)**	24 (25)	14 (26)	10 (23)	0.722
**Duration operative procedure (minutes)**	295 [227–360]	288 [216–353]	300 [242–374]	0.485
**Hospital stay (days)**	14 [9–25]	15 [10–26]	13 [8–22]	0.986
**Intraoperative RBC transfusion (units)**	0 [0–1]	0 [0–2]	0 [0–1]	0.489
**Intraoperative FFP transfusion (units)**	0 [0–4]	0 [0–4]	0 [0–4]	0.431
**CCI ^2^**	28 [0–47]	26 [26–48]	30 [0–47]	0.669

Values are given as the median and interquartile ranges or numbers and percentages. ^1^ Refers to Clavien et al. [17]. ^2^ Refers to Slankamenac et al. [18]. Abbreviations used: pCCA: perihilar cholangiocellular carcinoma, iCCA: intrahepatic cholangiocellular carcinoma, CD: Clavien–Dindo classification, RBC: red blood cell units, FFP: fresh frozen plasma units, CCI: comprehensive complication index.

## Data Availability

All relevant data were reported within the manuscript and Supplementary files. Further supporting data will be provided upon written request addressed to the corresponding author.

## Supplementary Materials

The following supporting information can be downloaded at: https://www.mdpi.com/article/10.3390/cancers17132213/s1, Table S1: Gender-specific carcinoma distribution.

## Abbreviations

ALT	Alanine aminotransferase
ASA	American Society of Anesthesiologists
AST	Aspartate aminotransferase
BC	Body composition
BMD	Bone mineral density
BMI	Body mass index
CCA	Cholangiocarcinoma
CCI	Comprehensive complication index
CD	Clavien–Dindo classification
CT	Computed tomography
FFP units	Fresh frozen plasma units
GCP	Good clinical practice
GGT	Gamma glutamyltransferase
HU	Hounsfield units
iCCA	Intrahepatic cholangiocarcinoma
ICU	Intensive care unit
L3	Third lumbar level
OLT	Orthotopic liver transplantation
OR	Odds-ratio
pCCA	Perihilar cholangiocarcinoma
PVE	Portal vein embolization
POD	Postoperative day
RBC units	Red blood cell units
SMA	Skeletal muscle radiation attenuation
SMI	Skeletal muscle index
UH-RWTH	University Hospital of the RWTH University
UICC	Union for International Cancer Control
