# Supplementary material for: Perioperative and Oncological Outcome in Patients Undergoing Curative-Intent Liver Resection for Cholangiocarcinoma in the Context of Osteopenia
Source: Cancers (Basel). 2025 Jul 1;17(13):2213. doi: 10.3390/cancers17132213 (PMC12248797; doi:10.3390/cancers17132213)
Supplement: Supplementary file 1 [file cancers-17-02213-s001.zip › cancers-3685674-supplementary.pdf]

Supplementary Table S1. Gender-specific carcinoma distribution.

**Characteristics**	**All patients**	**Osteopenia**	
**pCCA + iCCA**	**n=202**	**yes n=107**	**no n=95**
**Female n (%)**	86 (43)	44 (41)	42 (44)
**Male n (%)**	116 (57	63 (59)	53 (56)
**pCCA**	**n=105**	**yes n=54**	**no n=51**
**Female n (%)**	33 (31)	16 (30)	17 (33)
**Male n (%)**	72 (69)	38 (70)	34 (67)
**iCCA**	**n=97**	**yes n=53**	**no n=44**
**Female (n)**	53 (55)	28 (53)	25 (57)
**Male (n)**	44 (45)	25 (47)	19 (43)

Abbreviations used: pCCA: perihiliar cholangiocellular carcinoma, iCCA: intrahepatic cholangiocellular carcinoma
